# Synergistic Combination of Polydopamine and Polypyrrole in Natural Pectin/PVA-Based Freestanding Electrodes for High-Performance Supercapacitors

**DOI:** 10.1021/acsomega.4c10148

**Published:** 2025-02-06

**Authors:** Tzu-Yuan Yen, Jo-Ying Liu, Jincy Parayangattil Jyothibasu, Hongta Yang, Shan-Ho Chan, Hsiu-Li Lin, Yi-Ming Sun, Rong-Ho Lee

**Affiliations:** † Department of Chemical Engineering, National Chung Hsing University, Taichung 402, Taiwan; ‡ Department of Medical Imaging and Radiology, Shu-Zen Junior College of Medicine and Management, Kaohsiung 505, Taiwan; § Department of Chemical Engineering and Materials Science, Yuan Ze University, Taoyuan City 320, Taiwan

## Abstract

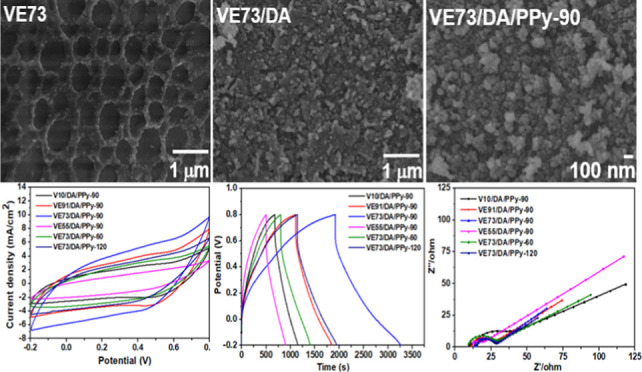

In this study, poly(vinyl alcohol) (PVA)/pectin/polypyrrole (PPy) and PVA/pectin/polydopamine (PDA)/PPy hydrogel films were prepared for use as supercapacitor electrodes. The synergistic effect of PDA and PPy on the electrochemical performance of the PVA/pectin/PDA/PPy hydrogel electrode was studied. PVA/pectin composite films (VE10, VE91, VE73, and VE55) with varying pectin proportions were prepared by cross-linking PVA with glutaraldehyde to serve as flexible and stretchable gel substrates for supercapacitor electrodes. PPy was synthesized on the surface of PVA/pectin films via chemical bath deposition. The incorporation of optimized pectin significantly enhanced the PPy content and the capacitance of the PVA/pectin/PPy film (VE73/PPy-90), achieving a measured value of 463.1 mF/cm^2^, which is notably higher than that of films without pectin. To further enhance the surface capacitance of the PVA/pectin/PPy composite, PDA was synthesized in situ on the surface of the PVA/pectin electrode using a chemical bath, followed by PPy polymerization. The synergistic combination of PDA and PPy resulted in a much higher areal and specific capacitance of 1575.7 mF/cm^2^ and 262.6 Fg^1–^ at a current density of 1 mA/cm^2^ for the PVA/pectin/PDA/PPy (VE73/DA/PPy-90) gel electrode. After charge–discharge cycle testing at a current density of 1 mA/cm^2^, the VE73/PPy-90 and VE73/DA/PPy-90 gel electrodes retained 80% and 70% of their initial capacitance, respectively, indicating reasonable cycle stability. The areal and specific capacitances of the symmetric supercapacitor based on the VE73/DA/PPy-90 electrode, estimated from the galvanostatic charge/discharge plots, were approximately 125.0 mF/cm^2^ and 20.8 F g^–1^, respectively, at a current density of 1 mA/cm^2^. The device exhibited moderate electrochemical stability, retaining 68% of its capacitance after 10,000 galvanostatic charge/discharge cycles at a current density of 1 mA/cm^2^.

## 1 Introduction

With the advent of new flexible and wearable electronic devices, the demand for energy storage solutions that are similarly flexible, stretchable, and adaptable has risen significantly. To meet this demand, researchers are exploring new ways to integrate energy storage devices into these products, while also satisfying their design requirements. One potential solution to this challenge is the development of flexible and stretchable supercapacitors, which offer lightweight, high flexibility, and good stretchability. However, traditional methods for preparing electrodes, such as slurry coating, often result in rigid structures that are not well-suited for use in flexible and stretchable devices. To overcome this challenge, researchers have focused on using polymer hydrogels, which can produce electrodes that are themselves flexible and stretchable. This alternative class of electrodes has the potential to enable the development of high-performance energy storage devices that are both flexible and stretchable.^1−5^

Biopolymer materials derived from natural plants are commonly used in the electrode and electrolyte materials of supercapacitors. For example, materials such as pectin, sunflower heads, and pomelo peels are often subjected to high-temperature carbonization processes to obtain porous carbon materials for electrode applications.^6,7^ Pectin are types of structural polysaccharides naturally occurring in the cell walls of plants. They consist of blocks of homogalacturonans and rhamnogalacturonans with neutral sugar side chains such as arabinose or rhamnose.^8,9^ Pectin can be classified into three categories, depending on the level of methyl esters present in the molecule: low-methoxylated pectin (LMP), medium-methoxylated pectin (MMP), and high-methoxylated pectin (HMP). While all pectin are capable of forming gels through a process known as ionotropic gelation, LMP and MMP can be cross-linked by multivalent cations, whereas HMP requires acidic conditions and the presence of solutes to form gels.^10^ Amarnath et al. reported the synthesis of polyaniline (PANI) using a pectin solution as a dispersant, which helped stabilize the dispersion of PANI particles and prevent aggregation. Silver ions were further reduced onto PANI to serve as electrodes for supercapacitors, albeit with relatively low capacitance values.^11^ In 2022, Gonzalez et al. reported the use of pectin as a dispersing and adhesive agent for graphene oxide (GO). GO was coated on a flexible substrate made from coconut fibers, and a layer of MgTiO_3_ dispersed in poly(methyl methacrylate) was further coated on top of GO to serve as electrodes for capacitors.^12^ Perumal and Selvin reported the preparation of a gel-like polymer by mixing water-soluble pectin with lithium bromide, which was then applied as an electrolyte for supercapacitors.^13^ Pectins have also been used as stabilizing agents in the aqueous synthesis of polypyrrole (PPy), where stable pectin/PPy dispersions were obtained. Pectin hydrogels were successfully prepared using calcium as the cross-linking agent, and the pectin/PPy composites have potential applications as electro-active hydrogels.^14^

Modifying the surface properties of polymeric substrates can be achieved by applying organic molecule coatings using covalent or noncovalent methods, offering the opportunity to introduce diverse functionalities. Among the noncovalent approaches, the utilization of polydopamine (PDA) chemistry has gained significant interest. The oxidation and self-polymerization of dopamine in an alkaline environment result in the formation of PDA. This versatile polymer can be used to coat a wide range of inorganic and organic substrates, allowing for precise control over the film’s thickness and ensuring excellent stability. Furthermore, the PDA coating possesses a high density of catechol and imine functionalities, making it a versatile and resilient platform for secondary reactions.^15^ This characteristic opens up opportunities for modifications tailored to specific applications, particularly within the realm of energy storage. Lee et al. employed a facile mussel-inspired surface modification technique to enhance the electrochemical performance of interconnected porous carbon nanosheet (IPCN) electrodes for high-performance electrochemical capacitors.^16^ The deposition of a PDA coating on IPCN electrodes led to a significant increase in specific capacitance by approximately 40%, attributed to the pseudocapacitance induced by the catechol groups in PDA. Coating the IPCN electrodes with PDA, as well as layers of Fe^3+^ and tannic acid, further enhanced the capacitance to approximately 244 F/g at 5 mV/s, an improvement of approximately 83% compared to unmodified IPCN electrodes, owing to the introduction of redox moieties and the strong interactions between Fe^3+^ ions and catechol groups.^16^ A facile and environmentally friendly electrosynthesis method was employed to create a PDA nanofilm supported on oxygen-functionalized carbon cloth (FCC). The surface functionalization of the carbon cloth facilitated strong adhesion of the PDA nanofilm, resulting in an electrode (PDA–FCC) with remarkable flexibility, an excellent electrical conductivity (22.6 mS), and superior wettability to the aqueous electrolyte. The PDA–FCC electrode exhibited a favorable capacitance of 617 mF/cm^2^ at 2.2 mA/cm^2^, showcasing excellent pseudocapacitive behavior attributed to the presence of catechol, amine, and imine moieties within the PDA.^17^

**Figure 1:**
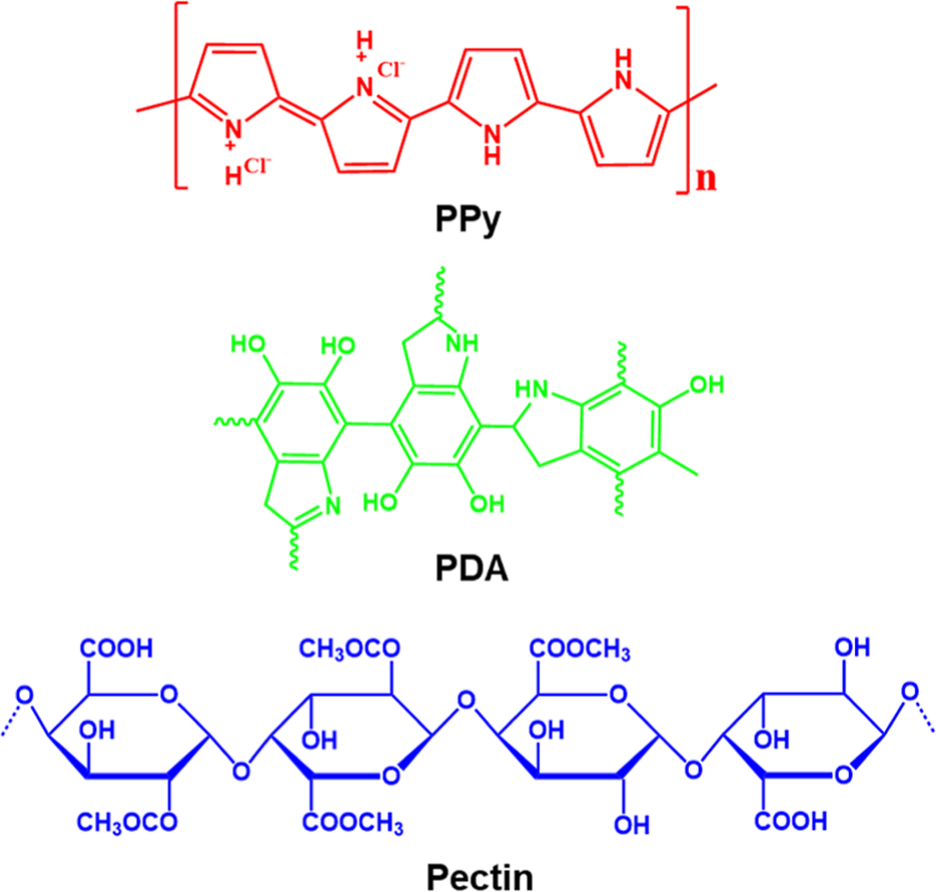
Chemical structures of PPy, PDA, and pectin.

In this study, PVA/pectin/PDA/PPy hydrogel composite films were prepared for use as flexible gel electrodes in supercapacitors. PVA is a synthetic polymer soluble in water and commonly used in supercapacitor applications due to its ease of preparation, good biodegradability, excellent chemical resistance, and strong mechanical properties.^18−21^ The chemical structures of pectin and PDA are shown in Figure 1. The blended PVA/pectin films combine the advantageous properties of both components to create advanced materials. Furthermore, the development of energy storage materials based on PVA and pectin aligns with the principles of sustainable development.^22^ Pectins were obtained from Aiyu seeds, also known as Creeping Figure seeds. Unlike other sources where pectin is primarily found inside the Aiyu seeds, pectins are concentrated in a transparent layer on the seed’s surface. Consequently, the extraction process involves washing and rubbing rather than grinding the seeds into a powder. It is worth noting that the resulting mucilaginous water extract from Aiyu seeds primarily consists of LMP, which differs from the HMP commonly found in commercially used sources such as apples or citrus peels.^23^ The PVA/pectin hydrogel films were fabricated through a chemical cross-linking process involving the reaction of poly(vinyl alcohol) (PVA) and pectin with glutaraldehyde (GA). This reaction occurs between the hydroxyl groups (–OH) of PVA and pectin and the aldehyde groups (–CHO) of GA, leading to the formation of acetal or hemiacetal bonds in a sulfuric acid solution.^24,25^ By utilization of this method, the PVA/pectin hydrogel films were successfully prepared, providing a robust and efficient electrode substrate. PDA and PPy were coated onto the PVA/pectin hydrogel films to impart electrochemical activity. The deposition of PDA and PPy onto the PVA/pectin hydrogel films was carried out by using soaking and polymerization techniques, ensuring uniform coverage and adhesion to the film surface. The presence of hydrogen bonding among the hydroxyl, amine, and carboxylic acid groups in PVA, pectin, PDA, and PPy leads to strong adhesion of PDA and PPy on the surface of the PVA/pectin film. The resulting PDA- and PPy-coated PVA/pectin hydrogel films offer enhanced electrochemical capabilities, making them suitable as supercapacitor electrodes. The presence of catechol groups in PDA provides redox-active entities that contribute to the pseudocapacitive behavior of the electrodes.^15−17^ The synergistic combination effect of PDA and PPy on the capacitance performance of the PVA/pectin/PDA/PPy electrodes was studied.

## 2 Experimental Section

### 2.1 Materials

Sulfuric acid, 3-hydroxytyramine, trishydrochloride, tris(hydroxymethyl)aminomethane, iron(III) chloride hexahydrate, pyrrole, PVA, and other chemicals were purchased from Sigma-Aldrich, J.T.Baker, Alfa Aesar, and Acros Organics and used as received.

### 2.2 Extraction of Pectin

The Aiyu seeds (*Ficus pumila* var. *awkeotsang*) were thoroughly washed in cold water to remove any dirt or debris. The cleaned seeds were then dried in an oven at 60 °C until constant weight was achieved. Ten g of Aiyu seeds were added to 600 mL of deionized water and stirred overnight at room temperature. The mixture was then filtered through a cheesecloth to remove the seeds and to collect the yellow-colored filtrate. The filtrate was then treated with 100 mL of ethanol (95%) to precipitate gelatinous pectin. The collected pectin was washed three times with ethanol to remove any impurities and dried on a heating plate. Finally, the dried Aiyu pectin was ground into a fine powder by using a ball mill to obtain the Aiyu pectin powder.

### 2.3 Preparation of PVA/Pectin Films

To prepare PVA/pectin films, pectin and PVA were accurately weighed and added to 8 mL of a 0.5 M sulfuric acid aqueous solution. The resulting mixture was treated in an ultrasonic shaker for 15 min to disperse the pectin powder. The mixture was then heated at 85 °C under stirring conditions on a magnetic stirrer for 1 h until a homogeneous solution was obtained. The solution was then cooled to 35 °C, and 0.8 mL of 1% glutaraldehyde was added under stirring conditions for the cross-linking reaction. The blend solution was quickly poured into a Petri dish and left at room temperature for 24 h for setting. The obtained gelatinous films were then placed in a beaker filled with DI water and washed until neutral to obtain PVA/pectin films. PVA/pectin films were dried by using the freeze-drying method. These blend films were prepared with different PVA/pectin ratios (10:0, 9:1, 7:3, and 5:5), while keeping the total weight of PVA and pectin in the composite film constant at 400 mg. The cross-linking time was dependent on the amount of PVA in the film, with higher PVA ratios leading to faster gel formation. The films made from blends of PVA and pectin were labeled according to their composition, with the ratios 10:0, 9:1, 7:3, and 5:5 corresponding to the names V10, VE91, VE73, and VE55, respectively. The weights of the V10, VE91, VE73, and VE55 films were 7.8, 6.2, 5.3, and 4.7 mg, respectively. The weights of the PVA/pectin composite films were decreased with increasing pectin content.

### 2.4 Preparation of PVA/Pectin/PPy Films

To fabricate the PVA/pectin/PPy film, a mixture of 2 mL of purified pyrrole (0.014 mol) and 1 mL of DI water was stirred in an ice bath for 30 min. Subsequently, a 1 cm × 1 cm PVA/pectin film was immersed in the mixture for 1 h to allow the pyrrole to absorb onto the film. The soaked film was then transferred into a 0.075 M FeCl_3_·6H_2_O aqueous solution to initiate the polymerization process, which lasted for 90 min. This process resulted in the formation of a PVA/pectin/PPy film. The film was purified by thoroughly washing it with DI water and centrifuging it to remove any remaining unreacted reagents and loosely bound PPy on its surface. The freeze-drying method was used to dry the PVA/pectin films. PPy-coated films of V10, VE91, VE73, and VE55 were abbreviated as V10/PPy-90, VE91/PPy-90, VE73/PPy-90, and VE55/PPy-90, respectively. The PPy contents in V10/PPy-90, VE91/PPy-90, VE73/PPy-90, and VE55/PPy-90 films were 14.5%, 26.2%, 40.5%, and 47.8%, respectively.

### 2.5 Preparation of PVA/Pectin/PDA Films

A Tris buffer with a pH value of 8.5 was prepared by dissolving 0.21 g of Trizma hydrochloride and 0.4 g of Trizma base in 100 mL of DI water. The pH of the buffer solution was confirmed by using a pH meter. To coat the PVA/pectin films with PDA, the films were immersed in the Tris buffer solution. Subsequently, 0.8 g of 3-hydroxytyramine was added to initiate the polymerization reaction. The reaction was carried out for 24 h under ambient conditions. After the reaction, the PDA-coated films were thoroughly cleaned by being rinsed with DI water to remove any excess reactants. The films were then centrifuged to remove loosely bound PDA from the PVA/pectin film. The PVA/pectin/PDA films were dried by using the freeze-drying method. PDA-coated films of V10, VE91, VE73, and VE55 were abbreviated as V10/DA, VE91/DA, VE73/DA, and VE55/DA, respectively. As the proportion of pectin increased, the amount of PDA anchored to the surface of the PVA/pectin composite films also increased. The PDA contents in V10/DA, VE91/DA, VE73/DA, and VE55/DA were 19.0%, 32.0%, 39.6%, and 51.0%, respectively.

### 2.6 Preparation of PVA/Pectin/PDA/PPy Films

Initially, a mixture of 2 mL of purified pyrrole (0.014 mol) and 1 mL of DI water was stirred in an ice bath for 30 min to ensure proper mixing. Next, a VE73/PDA film with dimensions of 1 cm × 1 cm was immersed in the mixture for 1 h, allowing the pyrrole molecules to be absorbed onto the film. After the absorption process, the soaked film was transferred to a 0.075 M FeCl_3_·6H_2_O aqueous solution to initiate the polymerization process. The film was left in the solution for the desired polymerization time. Once the polymerization reaction was complete, the films were carefully washed with DI water to remove any loosely bound PPy and unreacted reagents. To ensure thorough cleaning, the films were subjected to centrifugation. The freeze-drying method was used to dry the PVA/pectin/PDA/PPy films. PPy-coated films of V10/PDA, VE91/PDA, VE73/PDA, and VE55/PDA were abbreviated as V10/PDA/PPy-90, VE91/PDA/PPy-90, VE73/PDA/PPy-90, and VE55/PDA/PPy-90, respectively. The PVA/pectin/PDA/PPy films prepared with different polymerization times—specifically 60, 90, and 120 min—were labeled VE73/PDA/PPy-60, VE73/PDA/PPy-90, and VE73/PDA/PPy-120, respectively. The PDA contents in VE73/PDA/PPy-60, VE73/PDA/PPy-90, and VE73/PDA/PPy-120 were 39.0%, 39.6%, and 38.0%, respectively. The PPy contents in V10/PDA/PPy-90, VE91/PDA/PPy-90, VE73/PDA/PPy-90, VE55/PDA/PPy-90, VE73/PDA/PPy-60, and VE73/PDA/PPy-120 films were 7%, 16%, 21%, 26%, 6.2%, and 24.2%, respectively.

### 2.7 Chemical and Physical Analyses

Fourier transform infrared (FTIR) spectra were recorded by using a PerkinElmer FTIR spectrometer (Spectroscopy 100). Thermogravimetric analysis (TGA) was performed using a PerkinElmer Pyris 1 to determine the thermal decomposition temperatures (*T*_d_), which is defined as the temperature at which the composite films experience a 10% weight loss. TGA analysis was conducted in a nitrogen (N_2_) atmosphere with a heating rate of 10 °C min^–1^. The morphology of the composite films was examined using field emission scanning electron microscopy (FESEM, JSM 7401F; JEOL, Japan). Prior to SEM measurement, the composite films were freeze-dried for 24 h to remove any residual water.

The swelling ratio is an important parameter for evaluating the water absorption capacity and swelling performance of PVA/pectin/PPy, PVA/pectin/PDA, and PVA/pectin/PDA/PPy hydrogels. The swelling ratio refers to the ratio of the increased weight of a hydrogel after absorbing water to its original weight. This ratio can be calculated by measuring the weight of the hydrogel in both dry and swollen states using the following eq 1
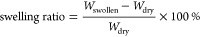
1where *W*_swollen_ is the weight of the hydrogel after absorbing water and *W*_dry_ is the weight of the hydrogel in the dry state.

The tensile test of PVA/pectin, PVA/pectin/PPy, PVA/pectin/PDA, and PVA/pectin/PDA/PPy flexible hydrogel specimens in this experiment is produced by pressing a standard-sized tensile mold (ASTM D412) onto the surface of the hydrogel film, forming it for the subsequent tensile test. Tensile testing was conducted using a testing machine (GOTECH, AI-3000). The tests were performed at a load speed of 10 mm/min. The conductivities of the hydrogel-based electrodes were measured by using a four-point probe resistance meter (Jiehan, SRS 4060).

### 2.8 Electrochemical Measurements

The PVA/pectin/PPy and PVA/pectin/PDA/PPy flexible hydrogel electrodes were evaluated for their electrochemical performance by using a three-electrode system on a CHI6273D electrochemical workstation (CH Instruments, Austin, TX, USA). The counter electrode was a platinum plate electrode, and a saturated calomel electrode was used as the reference electrode. All testing was conducted in a 1.0 M H_2_SO_4_ aqueous electrolyte. Cyclic voltammetry (CV) and galvanostatic charge/discharge (GCD) tests were conducted within the potential range from −0.2 to 0.8 V, while electrochemical impedance spectroscopy (EIS) measurements were obtained over the frequency range spanning from 0.01 Hz to 100 kHz.

The following eqs 2 and 3 were used to calculate the areal capacitance (*C*_a_, mF cm^–2^) and specific capacitance (*C*_s_, F g^–1^) of the composite electrodes^26,27^
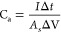
2
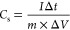
3where *I,* Δ*t, A*_*s*_, *m*, and Δ*V* denote the discharge current (*A*), the discharge time (*s*), the area of the freestanding electrode (cm^2^), the mass of the active material loaded on a single electrode (g), and the potential window (*V*), respectively.

The symmetric supercapacitor device was fabricated by sandwiching a filter paper separator soaked in 1 M Na_2_SO_4_ electrolyte between two identical pieces of PVA/pectin/PDA/PPy composite film-based gel electrodes. A platinum (Pt) plate was used as the current collector. Equations 4–6 were used to calculate the areal capacitance (*C*_cell_, F cm^–2^), energy density (*E*, μW h cm^–2^), and power density (*P*, μW cm^–2^) of the fabricated supercapacitor, respectively.^26,27^
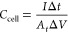
4
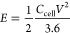
5
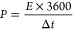
6where *I*, Δ*t*, Δ*V*, and *A*_*t*_ denote the discharge current (*A*), the discharge time (*s*), the potential window (*V*), and the geometric electrode working area (cm^2^), respectively.

## 3 Results and Discussion

### 3.1 Characterization of the PVA/Pectin/PPy and PVA/Pectin/PDA/PPy Composite Films

**Figure 2:**
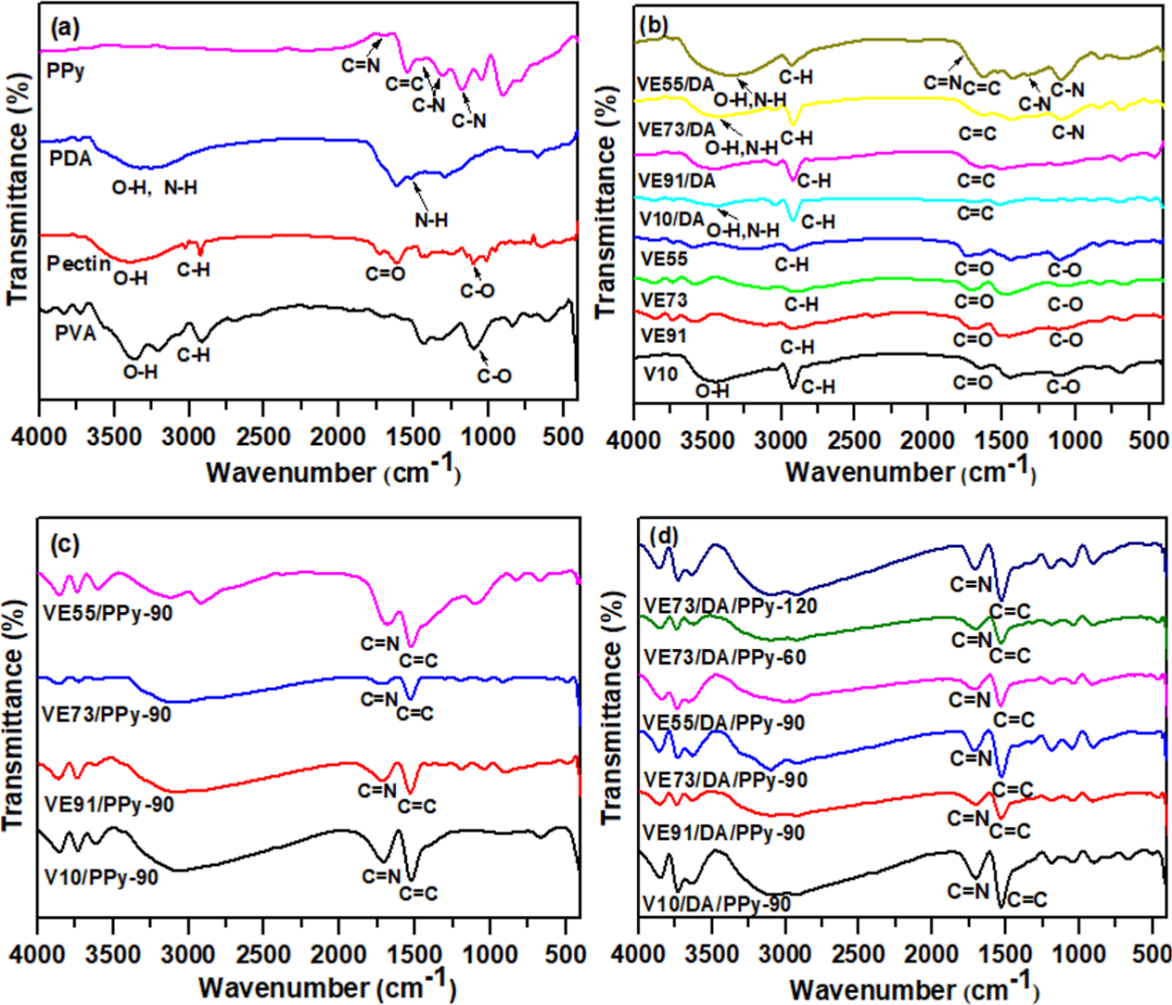
FTIR spectra of (a) PVA, pectin, PDA, and PPy; (b) V10, VE91, VE73, VE55, V10/DA, VE91/DA, VE73/DA, and VE55/DA; (c) V10/PPy-90, VE91/PPy-90, VE73/PPy-90, and VE55/PPy-90; and (d) V10/DA/PPy-90, VE91/DA/PPy-90, VE73/DA/PPy-90, VE55/DA/PPy-90, VE73/DA/PPy-60, and VE73/DA/PPy-120.

In Figure 2a, the broad peak observed in the range of 3000–3680 cm^–1^ for PVA corresponds to the intermolecular stretching vibrations of O–H. The shoulder peak between 2792 and 2986 cm^–1^ is attributed to the stretching vibrations of C–H. The peak at 1106 cm^–1^ corresponds to C–O vibrations. In the pectin spectrum, the broad peak between 3050 and3687 cm^–1^ is due to intermolecular hydrogen bonding-induced stretching vibrations of the O–H groups. The peaks at 1724 cm^–1^ and 1608 cm^–1^ can be attributed to the C=O stretching vibrations of the –COOH and –COO–R groups in pectin.^23,24^ For PDA, the broad peak observed in the range of 2990–3600 cm^–1^ is attributed to –NH and –OH stretching, while the peak at 1504 cm^–1^ is due to N–H stretching vibrations.^16^ Additionally, in the PPy spectrum, the peak at 1708 cm^–1^ is due to the C=N stretching vibrations. The band at 1538 cm^–1^ is due to the combination of the C=C and C–C stretching vibrations of PPy. The signal appearing at 1452 cm^–1^ is ascribed to C–N stretching vibrations, while the band at 1311 cm^–1^ represents the C–H and C–N in-plane deformation modes. The absorption bands near 1169 and 904 cm^–1^ indicate the presence of a doped PPy state. The absorption band at 1046 cm^–1^ is designated as the C–H in-plane deformation vibration of the PPy ring, while the signal at 790 cm^–1^ is due to the C–H out-of-plane ring deformations.^27^

In Figure 2b, the PVA/pectin composite films (V10, VE91, VE73, and VE55) show a significant reduction in the –OH region compared with the PVA spectrum. This reduction is attributed to cross-linking reactions between the hydroxyl groups of PVA and the aldehyde groups of GA, resulting in a notable decrease in peak intensity.^25^ For the V10 sample, the cross-linking reaction leads to the formation of ester groups, as indicated by a C=O stretching vibration peak at 1642 cm^–1^. This peak is observed for V10, which lacks pectin. In the cross-linked PVA/pectin-based films (VE91, VE73, and VE55), an additional C=O stretching vibration peak appears at 1727 cm^–1^, corresponding to the carbonyl groups of pectin.^23,24^ For the cross-linked PVA/pectin/PDA films, the presence of –OH groups in PDA causes a significant broadening of the intermolecular hydrogen-bonded –OH signals compared to the cross-linked PVA/Pectin films (Figure S1b). In Figure 2c,d, C=N and C=C vibration peaks from PPy are observed at approximately 1700 cm^–1^ and 1530 cm^–1^, respectively, for the PVA/pectin/PPy and PVA/pectin/PDA/PPy films, indicating successful synthesis of PPy on the hydrogel films.^27^

**Figure 3:**
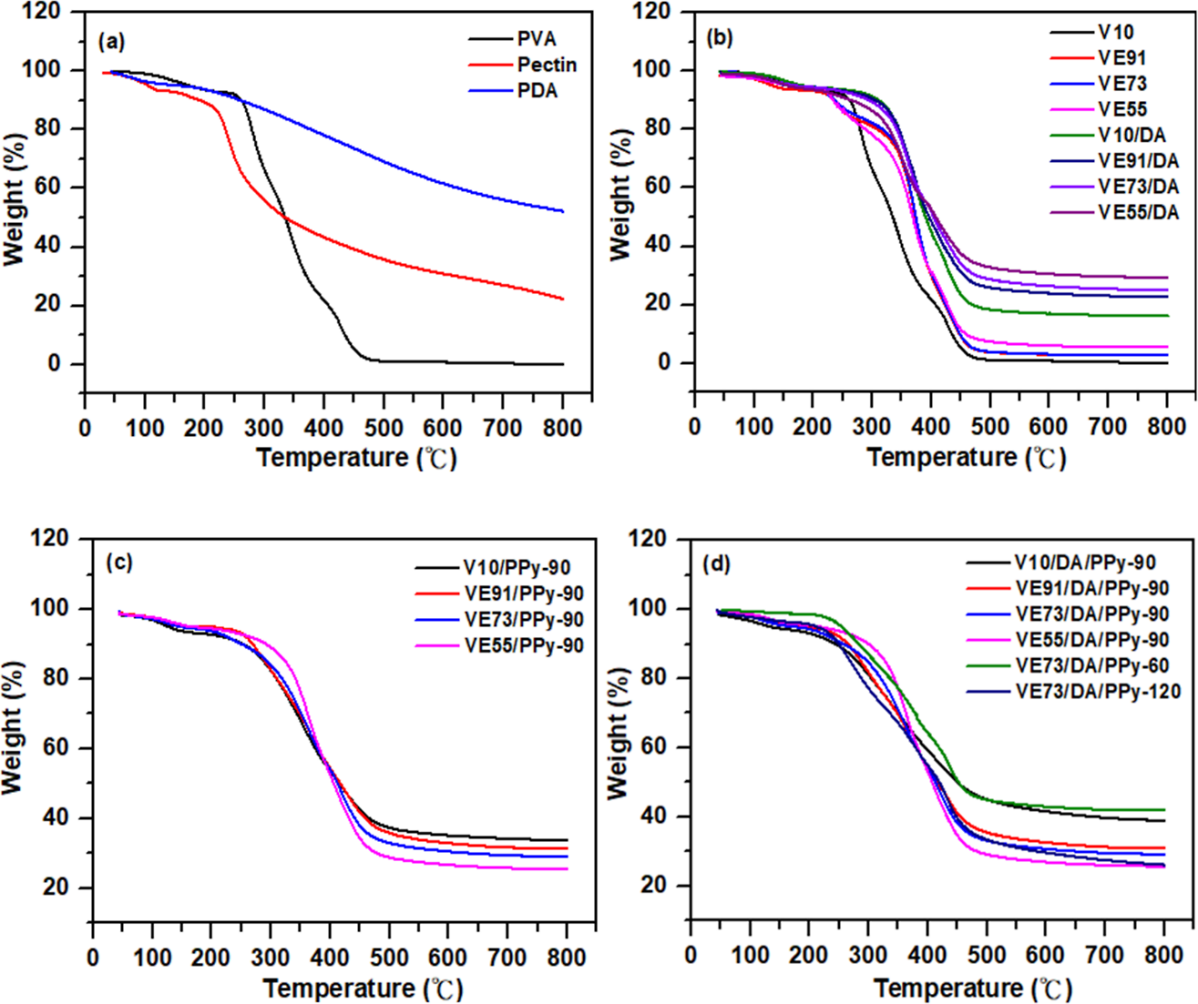
TGA thermograms of (a) pectin, PDA, and PVA; (b) V10, VE91, VE73, VE55, V10/DA, VE91/DA, VE73/DA, and VE55/DA; (c) V10/PPy-90, VE91/PPy-90, VE73/PPy-90, and VE55/PPy-90; and (d) V10/DA/PPy-90, VE91/DA/PPy-90, VE73/DA/PPy-90, VE55/DA/PPy-90, VE73/DA/PPy-60, and VE73/DA/PPy-120.

In Figure 3a, PVA exhibits two stages of weight loss. PVA pyrolysis can be considered as two steps; one between 200 and 320 °C for elimination reactions, followed by between 320 and 500 °C for chain scission and cyclization reactions.^28^ Moreover, the weight loss of pectin at temperatures below 150 °C was attributed to water evaporation. As the temperature gets higher than 200 °C, the thermal decomposition of pectin leads to additional weight loss, resulting in a residual char yield of 22.4%. In contrast, the weight loss of PDA increased with rising temperature. PDA with catechol and amino groups exhibited higher thermal stability and a larger residual char yield (52.2%) as compared to PVA and pectin. The thermal stabilities of the PVA/pectin hydrogel materials (V10, VE91, VE73, and VE55) are shown in Figure 3b. At temperatures above 300 °C, the weight values of the PVA/pectin hydrogel materials were higher than those of PVA alone, indicating the higher stability of pectin. Moreover, the PVA/pectin/PDA hydrogel materials exhibit higher thermal stability and char yields as compared to those of the PVA/pectin hydrogel materials. This improvement is attributed to the higher thermal stability and residual char yield of PDA at high temperatures relative to that of PVA and pectin. The char yields increased with increasing pectin content in the PVA/pectin/PDA hydrogel materials, which can be explained by the increased amount of anchored PDA on the surface of the PVA/pectin composite films as the proportion of pectin increased.

The thermal stability curves of the PVA/pectin/PPy hydrogel materials are shown in Figure 3c. Weight loss at temperatures below 200 °C is attributed to residual moisture in the gel and deprotonation. Above 200 °C, however, deprotonation of PPy releases hydrochloric acid, converting PPy into a carbon-rich alkaline form. This process, known as carbonization, releases significant amounts of gas, including hydrogen and chlorine.^29^ The residual char yields of the PVA/pectin/PPy films decrease with an increasing pectin content. This is because pectin forms hydrogen bonds with more pyrrole monomers, resulting in gel electrodes with increased PPy on the surface. Since PPy emits gases during thermal decomposition, gel electrodes with a higher PPy content tend to exhibit a lower residual char yield. Similarly, the residual char yields of the PVA/pectin/PDA/PPy films decrease with increasing pectin content, as seen with the PVA/pectin/PPy films (Figure 3d). For VE73/DA/PPy-60, VE73/DA/PPy-90, and VE73/DA/PPy-120, the residual char rates are 42.1%, 29.2%, and 26.1%, respectively. These results indicate that longer polymerization times for PPy lead to lower residual char yield, likely due to PPy’s lower thermal stability compared to PDA.^30^ Consequently, electrodes with a higher PPy content tend to lose more weight and exhibit a lower residual char yield.

### 3.2 Morphology of the PVA/Pectin/PPy and PVA/Pectin/PDA/PPy Composite Films

The cross-linking reaction of PVA was catalyzed with glutaraldehyde in a sulfuric acid aqueous solution, resulting in the formation of numerous pores on the surface of these gel electrodes.^2,31^ However, these surface pores shrink as water disperses from the PVA-based composite films, eventually leading to a surface without pores. Therefore, capturing the surface morphology requires drying the PVA/pectin composite films through freeze-drying to maintain the original shape of the pores as much as possible. Figure S1a shows the SEM surface morphology of the V10 composite film, while Figure S1b–d depicts the PVA/pectin composite films VE91, VE73, and VE55, respectively. PVA/pectin composite films exhibit large pores on the film surface, which decrease in size with an increasing pectin content. Figure S2a illustrates the SEM image of the V10/DA film, where the surface of the V10 film, without pectin, is covered with large spherical particles of PDA. The large pores on the surface of the PVA/pectin films were filled with the PDA particles. Additionally, Figure S2b–d shows that the density of PDA particles on the surface of the PVA/pectin/PDA composite films varies. As the proportion of pectin increases, more hydrogen bonds are formed between pectin and PDA, resulting in smaller and more evenly distributed PDA particles on the film surface. Moreover, a higher content of PDA was anchored on the surface of the PVA/pectin composite films with a higher pectin content. A small amount of aggregates was observed for the VE55/DA sample.

**Figure 4:**
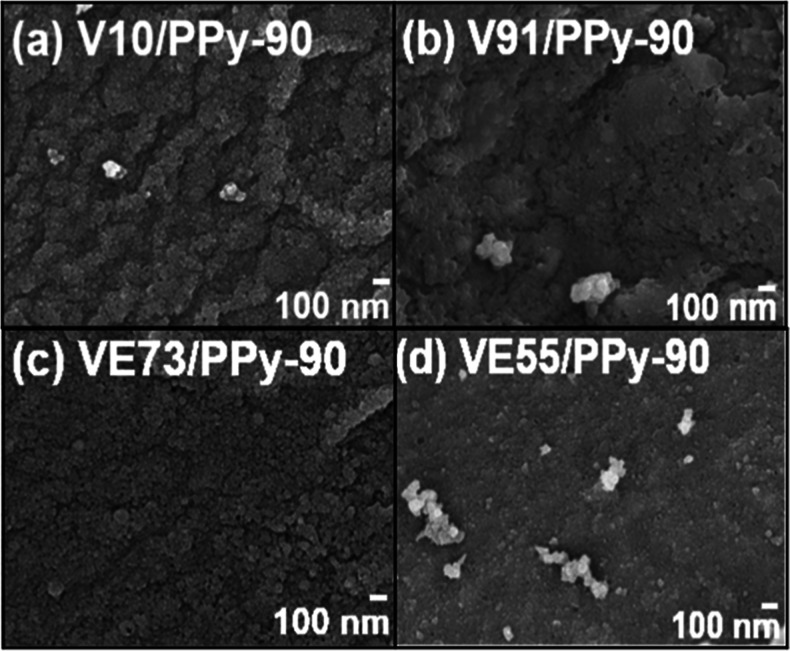
SEM images of the (a) V10/PPy-90, (b) VE91/PPy-90, (c) VE73/PPy-90, and (d) VE55/PPy-90 composite films.

Figure 4 depicts SEM images of PVA/pectin/PPy composite films (V10/PPy-90, VE91/PPy-90, VE73/PPy-90, and VE55/PPy-90). It can be observed that small spherical PPy particles (less than 100 nm) could not uniformly cover the pores on the surface of the PVA/pectin composite films for the V10/PPy-90 and VE91/PPy-90 samples. For these composite films, due to the lower pectin content, small PPy particles aggregated on the surfaces of the V10 and VE91 films, forming PPy-based domains with diameters of 200–300 nm. The low deposition amount of PPy could not smooth the surfaces of the V10 and VE91 composite films. In contrast, for the VE73/PPy-90 composite film, with increased pectin content, a larger amount of PPy particles polymerized on the VE73 surface, resulting in the most uniform and dense distribution of PPy particles. The surface of the PVA/pectin/PPy composite film became smooth after deposition with a large amount of PPy. However, PPy aggregates formed on the surface of the VE55/PPy-90 film. The hydrogen bonding between the carboxylic acid groups of pectin and the pyrrole groups of PPy promoted anchoring of the PPy particles on the surface of the PVA/pectin composite films.

**Figure 5:**
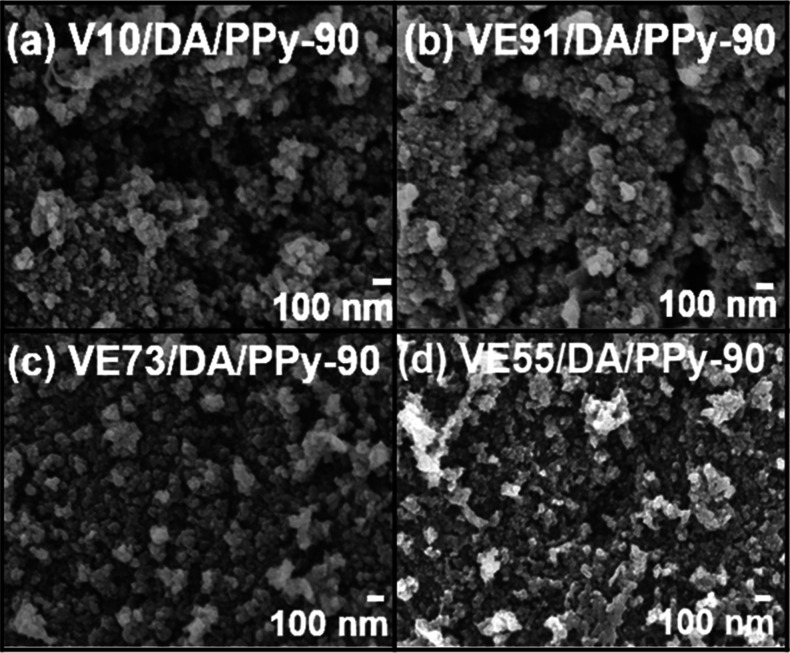
SEM images of the (a) V10/DA/PPy-90, (b) VE91/DA/PPy-90, (c) VE73/DA/PPy-90, and (d) VE55/DA/PPy-90 composite films.

SEM images of the PVA/pectin/PDA/PPy composite films (V10/DA/PPy-90, VE91/DA/PPy-90, VE73/DA/PPy-90, and VE55/DA/PPy-90) are shown in Figure 5. For the V10/DA/PPy-90 and VE91/DA/PPy-90 composite films, a small amount of PPy particles deposited on the surfaces of the V10/DA and VE91/DA films with large pores forming cracks. These pores and cracks disappeared for the VE73/DA/PPy-90 and VE55/DA/PPy-90 films, corresponding to the larger deposition amount of PPy. The PPy particles were uniformly distributed on the surface of VE73/DA/PPy-90. Nevertheless, some PPy aggregates were observed on the surface of the VE55/DA/PPy-90 film. In addition, the amount of PPy anchored on the PVA/pectin/PDA composite films was much lower than that on the PVA/pectin composite films, possibly due to the low specific surface area of the PDA-filled PVA/pectin/PDA composite films. The particle size of PPy on the PVA/pectin/PDA films was slightly larger than that on the PVA/pectin films. Furthermore, SEM images of the VE73/DA/PPy-60, VE73/DA/PPy-90, and VE73/DA/PPy-120 composite films are shown in Figure S3. The deposition amount of PPy on the surface of VE73/DA increased with an increasing PPy polymerization time. Small pores were observed on the surface of VE73/DA/PPy-60, which was attributed to the low deposition amount of PPy. In contrast, a large deposition amount of PPy led to the formation of cracks on the surface of VE73/DA/PPy-120.

### 3.3 Swelling Ratios of the PVA/Pectin/PPy and PVA/Pectin/PDA/PPy Composite Films

**Figure 6:**
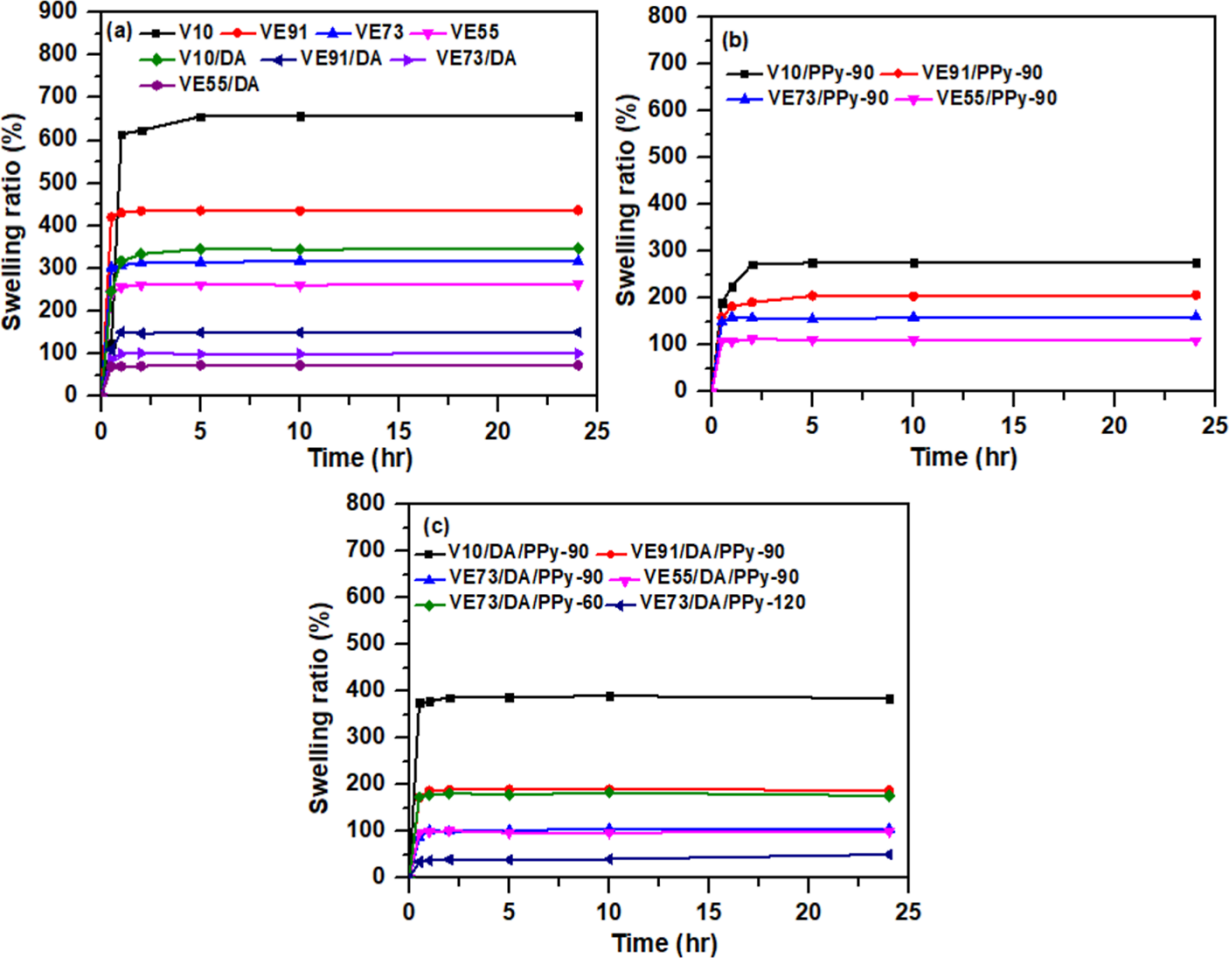
Swelling ratios of the (a) PVA/pectin and PVA/pectin/PDA, (b) PVA/pectin/PPy, and (c) PVA/pectin/PDA/PPy composite films.

For the PVA/pectin composite films, the cross-linking of PVA with glutaraldehyde leads to the formation of acetal bridges between the polymer backbones, weakening the hydrogen bonding between PVA and water. After soaking for 24 h, the swelling ratios of V10, VE91, VE73, and VE55 composite films are 657.4%, 436.9%, 316.5%, and 262.7%, respectively (Figure 6). The swelling ratios decrease with increasing pectin content in the PVA/pectin composite films, corresponding to the reduction of hydroxyl groups on the polymer chains of PVA. Additionally, the swelling ratios of V10/DA, VE91/DA, VE73/DA, and VE55/DA are 346.8%, 100.7%, 150.7%, and 73.1%, respectively. The swelling ratios of the PVA/pectin/PDA composite films are much lower than those of the PVA/pectin composite films. The deposition of the PDA layer on the surface of PVA/pectin composite films results in the reduction of hydrophilic groups on the surface of the composite films. As a result, the swelling ratios of the PVA/pectin/PDA composite films decrease. In Figure 6, the swelling ratios of the V10/PPy-90, VE91/PPy-90, VE73/PPy-90, and VE55/PPy-90 composite films are 276.3%, 206.3%, 160.3%, and 109.6%, respectively. The swelling ratios of the PVA/pectin/PPy composite films are much lower than those of the PVA/pectin composite films, attributed to the deposition of the low hydrophilicity of PPy. Figure S3 displays the swelling ratios of the PVA/pectin/PDA/PPy gel electrodes. The swelling ratios for VE10/DA/PPy-90, VE91/DA/PPy-90, VE73/DA/PPy-90, VE55/DA/PPy-90, VE73/DA/PPy-60, and VE73/DA/PPy-120 are 385.0%, 188.4%, 104.9%, 100.5%, 176.3%, and 50.7%, respectively. For the PVA/pectin/PDA/PPy composite films, the swelling ratios decrease with increasing pectin content, corresponding to the increase in PDA and PPy content on the surface of the composite films. The VE73/DA/PPy-120 composite film, with higher PPy content, exhibits a higher swelling ratio than those of VE73/DA/PPy-60 and VE73/DA/PPy-90. As shown in Figure S3c, a large deposition amount of PPy led to the formation of cracks on the surface of VE73/DA/PPy-120, which is favorable for the permeation of water into the PPy layer.

### 3.4 Tensile Strengths of the PVA/Pectin/PPy and PVA/Pectin/PDA/PPy Composite Films

The tensile stress–strain curves of the PVA/pectin composite films are shown in Figure S4a. The tensile stress of V10, VE91, VE73, and VE55 are 5.20, 2.37, 0.11, and 0.04 MPa, while the elongations at break are 216.1, 221, 124.9, and 94.7%, respectively. The tensile stress and elongation values at breaks were both decreased with increasing pectin content, which was attributed to the decreased PVA content and cross-linking density. Low tensile strength and Young’s modulus were observed for the VE73 and VE55 composite films. In Figure S4b, tensile stress and elongation at breaks of the PVA/pectin/PDA composite films were decreased as compared to the PVA/pectin composite films. The formation of hydrogen bonding between PDA and PVA/pectin film results in the decrease of the physical cross-linking between PVA and pectin. Therefore, the tensile stress and elongation at breaks were decreased for the PVA/pectin/PDA composite films. Nevertheless, the tensile strength and Young’s modulus of VE73/DA and VE55/DA were higher than those of VE73 and VE55, respectively. This was attributed to the high rigidity of catechol groups in PDA. In addition, lower tensile stress and elongation at breaks were observed for the PVA/pectin/PPy composite films as compared to the PVA/pectin/PDA composite films (Figure S4c). This is because of the higher flexibility and lower rigidity of the PPy. Higher deposition content of PPy led to the higher tensile strength and Young’s modulus of VE73/PPy-90 and VE55/PPy-90 as compared to those of V10/PPy-90 and VE91/PPy-90. However, the elongation at break was decreased significantly for the PVA/pectin/PPy composite film with a high pectin content. For the PVA/pectin/PDA/PPy composite films, the VE73/DA/PPy-90 and V55/DA/PPy-90 samples exhibited higher tensile strength and lower elongation than those of the VE10/DA/PPy-90 and V91/DA/PPy-90 samples (Figure S4d). Moreover, VE73/DA/PPy-90 shows higher tensile strength than VE73/DA/PPy-60 and VE73/DA/PPy-120, while the larger elongation was observed for VE73/DA/PPy-60.

### 3.5 Electrochemical Properties of the PVA/Pectin/PPy and PVA/Pectin/PDA/PPy Composite Film-Based Electrodes

**Figure 7:**
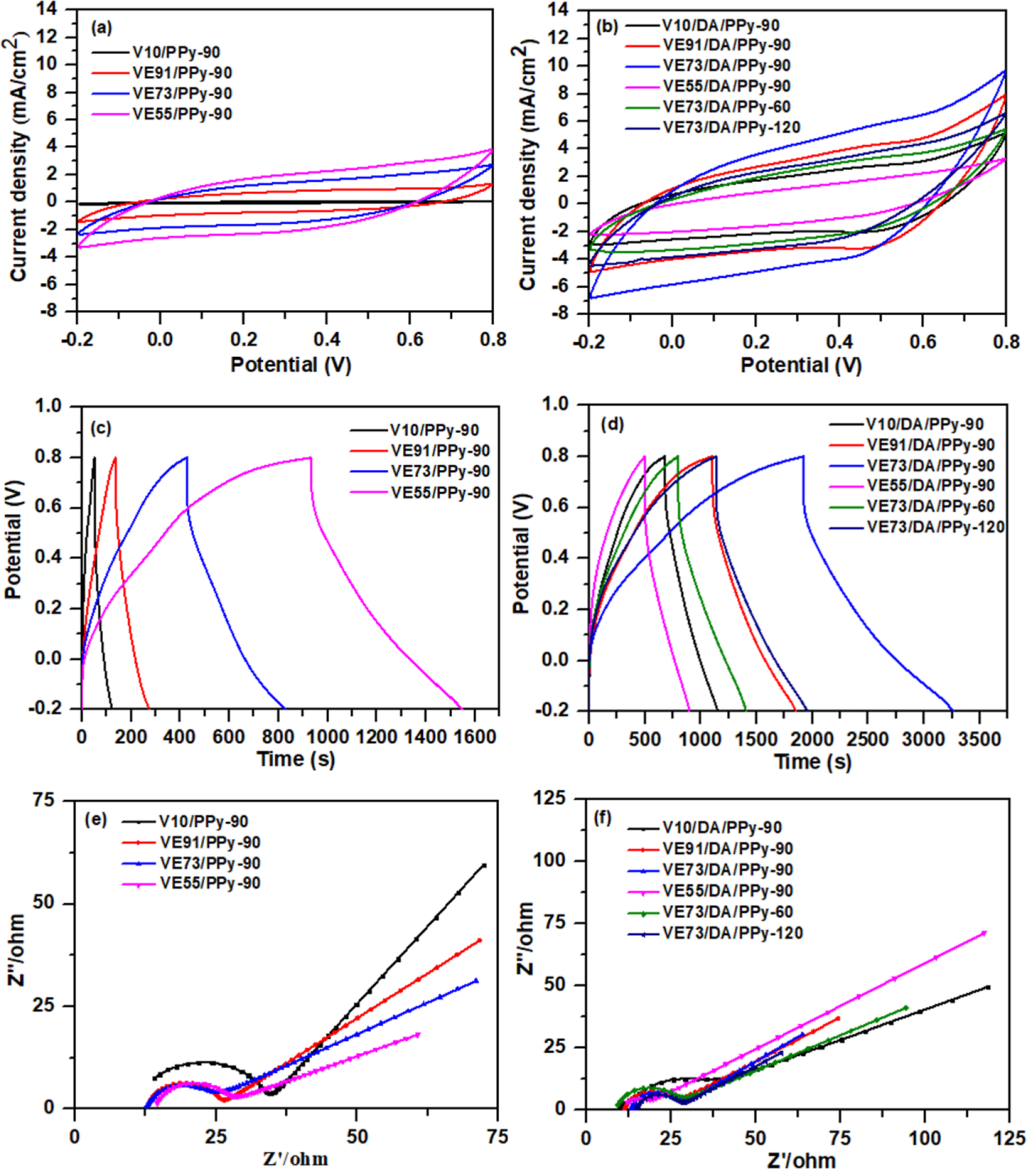
Electrochemical performances of the PVA/pectin/PPy and PVA/pectin/PDA/PPy composite films [(a,b) CV plots (scan rate: 5 mV s^–1^), (c,d) GCD plots (current density: 1 mA cm^–2^), and (e,f) EIS].

The electrochemical properties of the composite electrodes were evaluated in the potential range of −0.2 to 0.8 V using a three-electrode setup. The CV curves of the PVA/pectin/PPy composite electrodes at 5 mV s^–1^ are shown in Figure 7a. The capacitance of the electrodes was derived from the pseudocapacitance of the PPy. The PVA/pectin/PPy composites exhibited pseudocapacitance generated by the rapid and reversible charge-transfer reactions occurring on the surface of PPy. Since the capacitance characteristic is proportional to the area enclosed by the CV curve, the areas of the CV curves for the PVA/pectin/PPy composite electrodes increased with pectin content in the composite films, corresponding to the enhancement of the anchored content of PPy. The largest CV curve area was observed for the VE55/PPy-90 electrode at the same scan rate.

Additionally, the areas of the CV curves for the PVA/pectin/PDA/PPy composite electrodes exhibited significant enlargement as compared to those of the PVA/pectin/PPy composite electrodes, as depicted in Figure 7b. The capacitance of the electrodes was derived from the pseudocapacitance contributions of both PDA and PPy. A small oxidation peak was observed at approximately 0.5 V. The synergistic combination of higher conductivity of PDA and larger redox-active property of PPy led to larger capacitance values in the PVA/pectin/PDA/PPy composite electrodes, despite the lower PPy content as compared to the PVA/Pectin/PPy composite electrodes. The VE73/DA/PPy-90 composite electrode, with the largest enclosed CV area, provides the best capacitance characteristic among all the electrodes. The CV plots of the VE73/DA/PPy-90 electrode at different scan rates are shown in Figure S5a. The CV area of VE55/DA/PPy-90 was smaller than that of VE73/DA/PPy-90, which might be due to the aggregation of PPy particles in the VE55/DA/PPy-90 electrode. Moreover, the CV area of the VE73/DA/PPy-90 electrode was larger than those of the VE73/DA/PPy-60 and VE73/DA/PPy-120 electrodes. The optimized content of PPy on the surface of the PDA layer led to the better capacitance performance of the VE73/DA/PPy-90 electrode.

The GCD plots of the composite electrodes measured at 1 mA cm^–2^ are shown in Figure 7c,d. The PVA/pectin/PDA/PPy electrodes had a significantly longer discharge time than the PVA/pectin/PPy electrodes, indicating a significantly higher specific capacitance due to the synergistic effects of PDA and PPy, consistent with the CV results. We attribute the IR drops observed in the discharge curves of the PVA/pectin/PPy and PVA/pectin/PDA/PPy electrodes to the relatively high resistance of the hydrogel-based electrodes. The areal capacitances of the V10/PPy-90, VE91/PPy-90, VE73/PPy-90, VE55/PPy-90, V10/DA/PPy-90, VE91/DA/PPy-90, VE73/DA/PPy-90, VE55/DA/PPy-90, VE73/DA/PPy-60, and VE73/DA/PPy-120 composites were 81.1, 153.8, 463.1, 615.2, 504.6, 826.3, 1575.7, 467.2, 667.2, and 991.0 mF cm^–2^, respectively, based on their GCD plots measured at 1 mA cm^–2^. Moreover, the specific capacitances of the V10/PPy-90, VE91/PPy-90, VE73/PPy-90, VE55/PPy-90, V10/DA/PPy-90, VE91/DA/PPy-90, VE73/DA/PPy-90, VE55/DA/PPy-90, VE73/DA/PPy-60, and VE73/DA/PPy-120 composites were 67.6, 69.9, 128.6, 143.1, 188.8, 192.2, 262.6, 55.6, 158.9, and 152.5 F g^–1^, respectively, based on their GCD plots measured at 1 mA cm^–2^. The areal and specific capacitances increased with pectin and PPy contents in the PVA/pectin/PPy composite electrodes. The capacitances of the PVA/pectin/PDA/PPy composite electrodes exhibited greater values in comparison to those of the PVA/pectin/PPy composite electrodes. The VE55/DA/PPy-90 composite demonstrated a decrease in capacitance compared with the VE73/DA/PPy-90 composite. This can be attributed to the higher deposition content and aggregation of PPy on the PDA surface, as depicted in Figure 5c,d. Moreover, the capacitance of the VE73/DA/PPy-90 electrode was larger than those of the VE73/DA/PPy-60 and VE73/DA/PPy-120 electrodes. The GCD plots of the VE73/DA/PPy-90 electrode at different current densities are shown in Figure S5b. The aggregation and crack formation from a large amount of PPy deposited on the surface of the PDA layer in the VE73/DA/PPy-120 electrode (Figure S3c) resulted in its capacitance being smaller than that of the VE73/DA/PPy-90 electrode. The areal and specific capacitances obtained for the VE73/DA/PPy-90-based electrode were comparable to or superior to those of other recently reported PVA-based hydrogel electrodes (Table S1).^1,2,18,32−37^

EIS is one of the essential factors to be examined for the internal resistance and capacitive behavior of supercapacitors. Figure 7e,f shows the Nyquist plots of V10/PPy-90, VE91/PPy-90, VE73/PPy-90, VE55/PPy-90, V10/DA/PPy-90, VE91/DA/PPy-90, VE73/DA/PPy-90, VE55/DA/PPy-90, VE73/DA/PPy-60, and VE73/DA/PPy-120 composites. The Nyquist plots exhibit a semicircle in the high-frequency region and an oblique line in the low-frequency region, corresponding to the charge-transfer resistance (*R*_ct_) and Warburg diffusion impedance (*R*_d_), respectively.^38^ The *R*_ct_ of active electrode materials was determined from the diameter of the semicircle in the high-frequency region. The equivalent series resistance (*R*_s_) was calculated from the intersection on the real component (Z′) of the EIS plots in the high-frequency region. The *R*_ct_, *R*_d_, and *R*_s_ are related to the internal resistance of the active material, the ionic resistance of the electrolyte, and the contact resistance at the interface between the active material and the current collector, respectively.^27^ In Figure 7e, lower *R*_ct_ values were observed for the PVA/pectin/PPy electrodes with a higher PPy content, excluding the V10/PPy-90 electrode. This was attributed to the higher redox capacity of the electrodes. The *R*_s_ values slightly increased with increasing PPy content for the electrodes. Moreover, *R*_d_ increased with increasing PPy content, which corresponds to the dense packing density of PPy particles. Additionally, the *R*_ct_ values of the PVA/pectin/PDA/PPy electrodes decreased with an increasing PPy content. The VE73/DA/PPy-90 electrode exhibited the smallest *R*_ct_ value as compared with the V10/DA/PPy-90, V91/DA/PPy-90, and V55/DA/PPy-90 electrodes. Furthermore, the *R*_ct_ value of the VE73/DA/PPy-90 electrode was smaller than those of the VE73/DA/PPy-60 and VE73/DA/PPy-120 electrodes. The aggregation of the PPy particles results in an increase of the *R*_ct_ value of the VE73/DA/PPy-120 electrode. The *R*_s_ values slightly increased with the PPy content for the electrodes. In addition, the *R*_d_ values of the PVA/pectin/PDA/PPy electrodes slightly decreased with an increase in PPy content. As compared to the PVA/pectin/PPy electrodes, the synergistic combination of higher conductivity of PDA and larger redox-active property of PPy results in lower resistances of the PVA/pectin/PDA/PPy electrodes, especially for the VE73/DA/PPy-90 electrode. The conductivities of the PVA/pectin/PDA/PPy electrodes were further verified using a four-point probe resistance meter. The conductivities of V10/DA/PPy-90, VE91/DA/PPy-90, VE73/DA/PPy-90, and VE55/DA/PPy-90 were measured as 1.21, 3.26, 6.25, and 4.53 S·cm^–1^, respectively. The increase in the conductivity corresponds to the higher PPy content.

**Figure 8:**
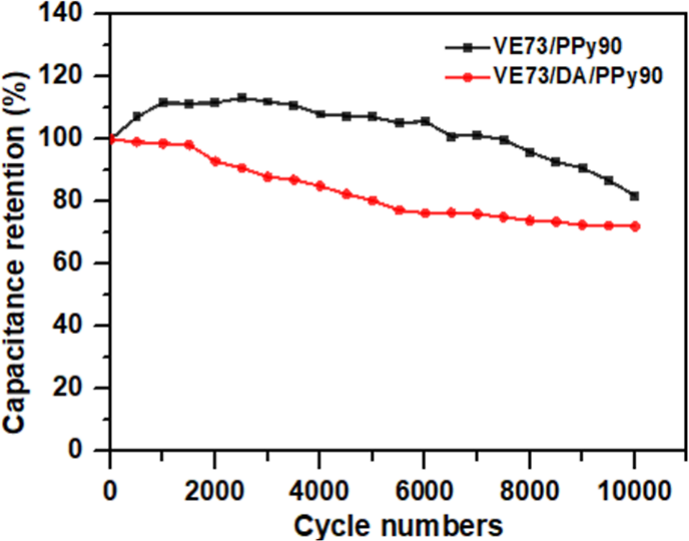
Cycling performance of the VE73/PPy-90 and VE73/DA/PPy-90 electrodes, recorded at 1 mA cm^–2^.

Since cycling stability is an important criterion for electrode materials, VE73/PPy-90 and VE73/DA/PPy-90 electrodes were charged and discharged 10,000 times at a current density of 1 mA cm^–2^ to measure the cycling performance. In Figure 8, for the VE73/PPy-90 electrode, it was observed that the areal capacitance did not decrease initially; instead, it increased. The areal capacitance began to decrease only after 3000 cycles. This may be due to the higher swelling ratio of the gel electrode at this proportion (approximately 150%, reaching equilibrium after 1 h), requiring more time for the electrode to become fully saturated with the sulfuric acid electrolyte. As the contact area between the electrode and electrolyte increases, the capacitance also increases. Even after 10,000 charge–discharge cycles, the areal capacitance retained 80% of its original value. In contrast, in the VE73/DA/PPy-90 electrode, the dense packing of the PDA layer on the surface of the VE73 film resulted in a lower swelling ratio (approximately 90%, reaching equilibrium after 0.5 h). Consequently, there was no initial increase, followed by a decrease in areal capacitance. The fluffy structure of the PPy layer also led to poorer cycling stability with only 70% of the original areal capacitance remaining. This is because the redox reactions during the charge–discharge cycles cause repeated expansion and contraction of the PPy structure.^39^ The cycling stability obtained for the VE73/DA/PPy-90-based electrode was comparable to those of other recently reported PVA-based hydrogel electrodes (Table S1).^1,2,18,32−37^

### 3.6 Electrochemical Properties of the PVA/Pectin/PDA/PPy Composite Film-Based Symmetric Supercapacitor

**Figure 9:**
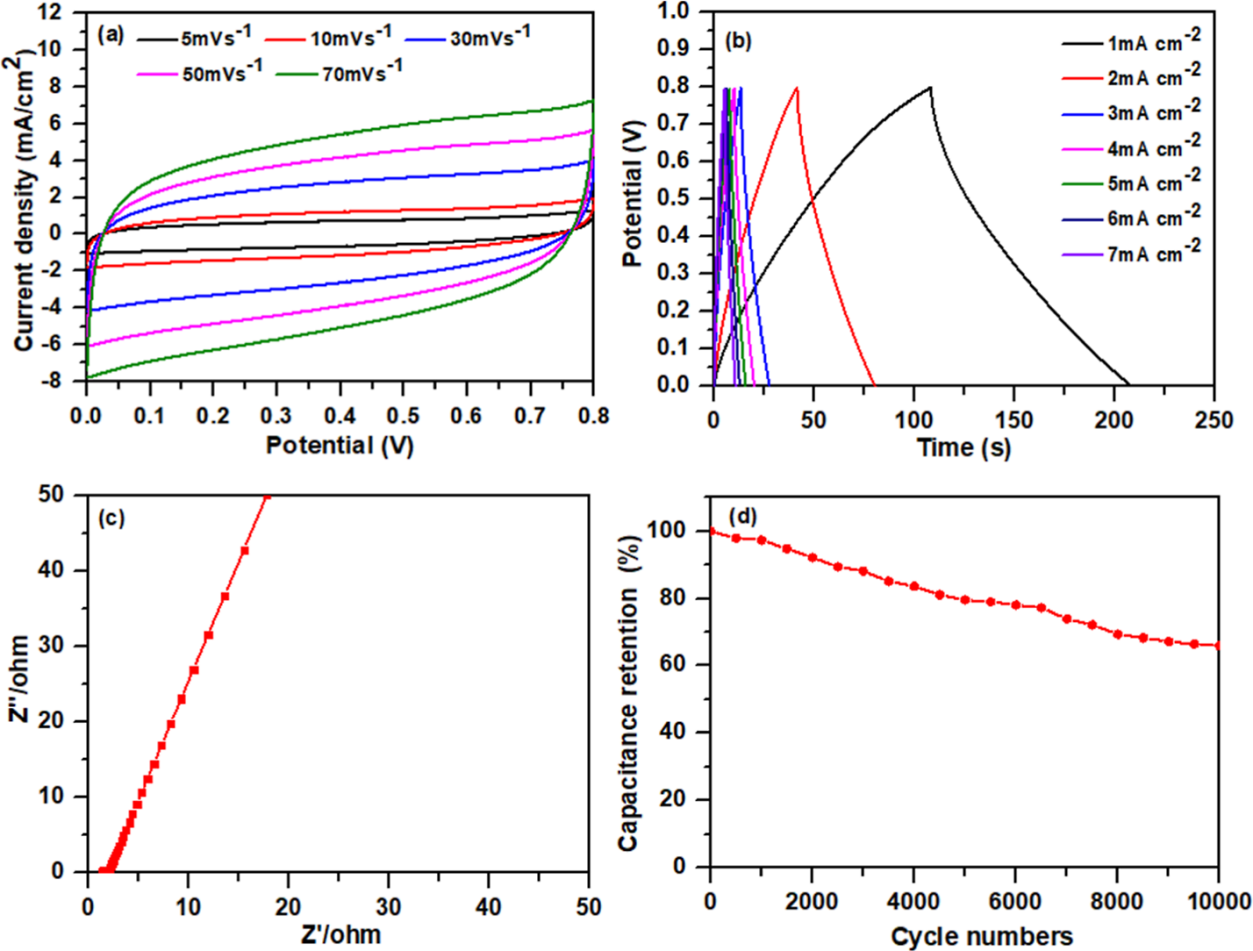
(a) CV plots at different scan rates, (b) GCD plots at different current densities, (c) EIS, and (d) cycling performance of the VE73/DA/PPy-90 electrode-based supercapacitor.

**Figure 10:**
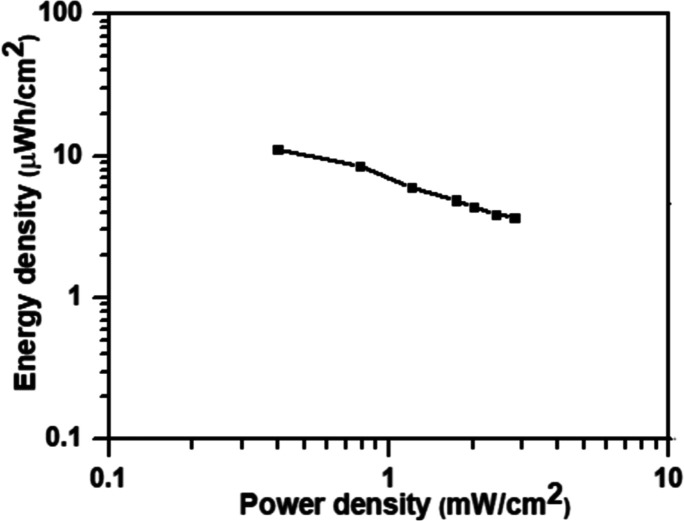
Ragone plot of the VE73/DA/PPy-90 electrode-based supercapacitor.

A symmetric supercapacitor device was constructed using VE73/DA/PPy-90 composite electrodes, a filter paper separator soaked in 1 M Na_2_SO_4_ (electrolyte), and a nickel foam current collector, as shown in Figure 9a. The supercapacitor exhibited slightly distorted rectangular CV profiles [Figure 9b] and symmetric triangular GCD plots [Figure 9c]. The areal and specific capacitances of the supercapacitor cell, estimated from the GCD plots, were approximately 125.0 mF cm^–2^ and 20.8 F g^–1^, respectively, at a current density of 1 mA cm^–2^. From the Nyquist plot [Figure 9d], a negligible semicircle was observed in the high-frequency region, indicating a low equivalent series resistance (*R*_s_) of 1.33 Ω and a charge-transfer resistance (*R*_ct_) of 0.11 Ω, confirming the efficient charge-transfer kinetics of the VE73/DA/PPy-90 electrode-based supercapacitor.^35^ Compared to the VE73/DA/PPy-90 electrode alone, lower resistances were observed in the supercapacitor, which corresponded to the use of current collectors. The adhered metal current collectors were able to make full contact with the active materials in the electrodes, significantly reducing the internal impedance. As a result, the *R*_s_ and *R*_ct_ values decreased. The cycle life of the assembled device was examined through 10,000 GCD cycles. Figure 7d shows the plot of the capacitance retention over the cycle number. The device exhibited moderate electrochemical stability, with 68% capacitance retention after 10,000 GCD cycles at a current density of 1 mA cm^–2^. Figure 10 displays the Ragone plot of the VE73/DA/PPy-90 electrode-based supercapacitor. The supercapacitor demonstrated a maximum areal energy density of 11.1 μW h cm^–2^ at a power density of 0.4 mW cm^–2^. Furthermore, the cell maintained an energy density of 3.7 μW h cm^–2^ at a power density of 2.8 mW cm^–2^. The energy density and power density of the VE73/DA/PPy-90 electrode-based supercapacitor were comparable or superior to other recently reported supercapacitors, such as the PANI@Ti_3_C_2_Tx/PVA hydrogel electrode-based supercapacitor,^18^ the PEDOT/PVA hydrogel electrode-based supercapacitor,^36^ and the PPy/PVA hydrogel electrode-based supercapacitor.^37^

## 4 Conclusions

PVA/Pectin hydrogel composite films with varying pectin proportions were prepared by cross-linking PVA with glutaraldehyde to serve as flexible and stretchable gel substrates for supercapacitor electrodes. PPy was deposited on the surface of the PVA/pectin composite films via chemical bath deposition. The hydrogen bonding between the carboxylic acid groups of pectin and the pyrrole groups of PPy promoted the anchoring of PPy particles on the surfaces of the PVA/pectin composite films. As a result, the amount of PPy anchored to the surface of the PVA/pectin composite films increased with a higher pectin content. With increased pectin content, more PPy particles polymerized on the surface of the PVA/pectin film, resulting in a uniform and dense distribution of PPy particles. The incorporation of pectin significantly improved the capacitance of the PVA/pectin/PPy composite film, which was notably higher than that of films without pectin. Furthermore, PDA was synthesized in situ on the surface of the PVA/pectin electrode using a chemical bath, followed by PPy polymerization. The amount of PPy anchored to the surface of the PVA/pectin/PDA film was lower than that on the PVA/pectin film. However, the synergistic combination of the higher conductivity of PDA and the greater redox-active properties of PPy led to larger capacitance values in the PVA/pectin/PDA/PPy composite electrodes despite the lower PPy content as compared to the PVA/pectin/PPy composite electrodes. Additionally, after 10,000 charge–discharge cycles, the VE73/PPy-90 and VE73/DA/PPy-90 gel electrodes display reasonable cycle stability. The cycle stabilities of the PVA/pectin/PPy and PVA/pectin/PDA/PPy hydrogel electrodes were strongly dependent on the dense packing or the fluffy structure of the PPy layer. The symmetric supercapacitor fabricated from the VE73/DA/PPy-90 electrode exhibited a moderate areal capacitance and electrochemical stability.

## Supporting Information Available

SEM images of the PVA/pectin films; SEM images of the PVA/pectin/PDA films; SEM images of the VE73/DA/PPy films; stress–strain curves of the PVA/pectin/PPy and PVA/pectin/PDA/PPy films; and capacitive performances of PVA-based hydrogel electrodes (PDF)
