# Supplementary material for: Synergistic Combination of Polydopamine and Polypyrrole in Natural Pectin/PVA-Based Freestanding Electrodes for High-Performance Supercapacitors
Source: ACS Omega. 2025 Feb 6;10(6):6025–37. doi: 10.1021/acsomega.4c10148 (PMC11840602; doi:10.1021/acsomega.4c10148)
Supplement: Supplementary file 1 — ao4c10148_si_001.pdf [file ao4c10148_si_001.pdf]

Supporting Information

Synergistic combination of polydopamine and polypyrrole in natural pectin/PVA-based freestanding electrodes for high-performance supercapacitors

Tzu-Yuan Yen[1§], Jo-Ying Liu[1§], Jincy Parayangattil Jyothibasu[1], Hongta Yang[1], Shan-Ho Chan[2], Hsiu-Li Lin[3], Yi-Ming Sun[3], and Rong-Ho Lee[1, 3*]

1. Department of Chemical Engineering, National Chung Hsing University, Taichung 402, Taiwan
2. Department of Medical Imaging and Radiology, Shu-Zen Junior College of Medicine and Management, Kaohsiung, Taiwan.
3. Department of Chemical Engineering and Materials Science, Yuan Ze University, Taoyuan City 320, Taiwan

*To whom correspondence should be addressed.

§ These authors contributed equally to this work

Rong-Ho Lee—e-mail: rhl@dragonnchu.edu.tw; tel.: +886-4-22854308; fax: +886-4-22854734.

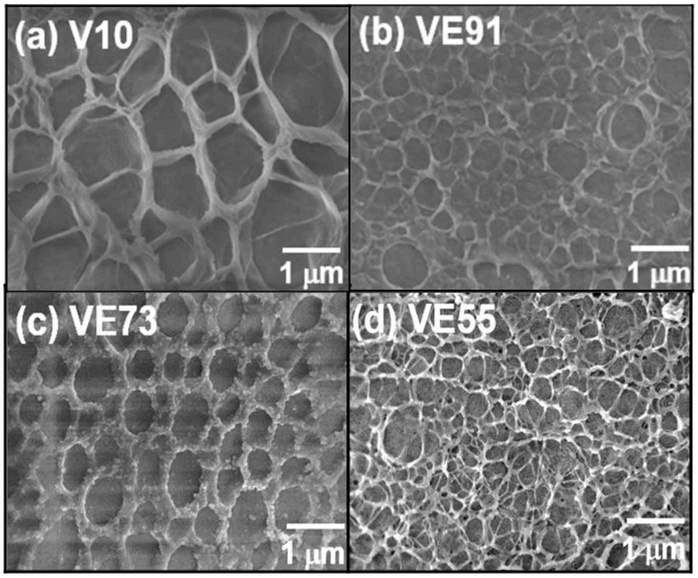

Figure S1. SEM images of the (a) V10,(b) VE91, (c) VE73, and (d) VE55 composite films.

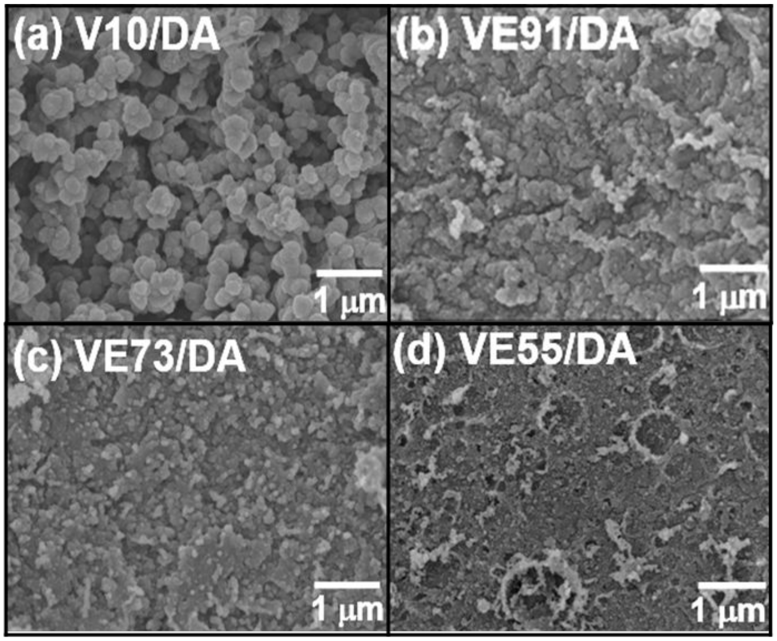

Figure S2. SEM images of the (a) V10/DA, (b) VE91/DA, (c) VE73/DA, and (d) VE55/DA composite films.

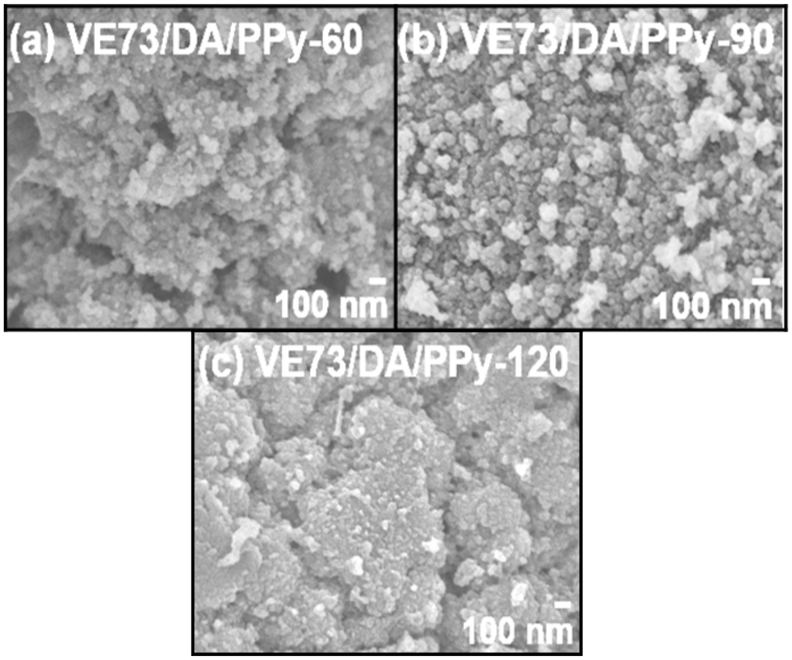

Figure S3. SEM images of the (a) VE73/DA/PPy-60, and (b) VE73/DA/PPy-90, and (c) VE73/DA/PPy-120 composite films.

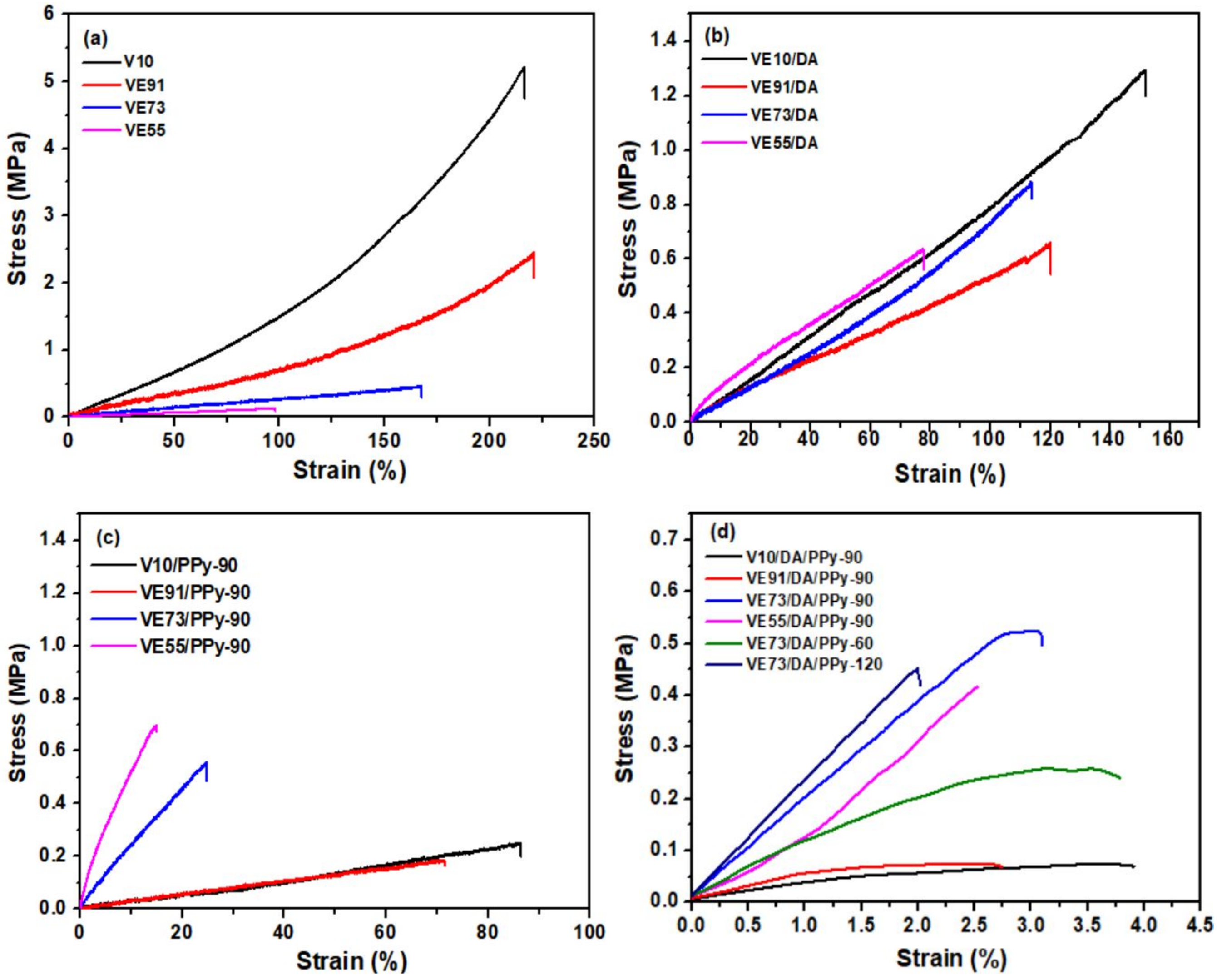

Figure S4 Stress-strain curves of the (a) PVA/pectin, (b) PVA/Pctin/PDA, (c) PVA/Pectin/PPy, and (d) PVA/Pectin/PDA/PPy composite films.

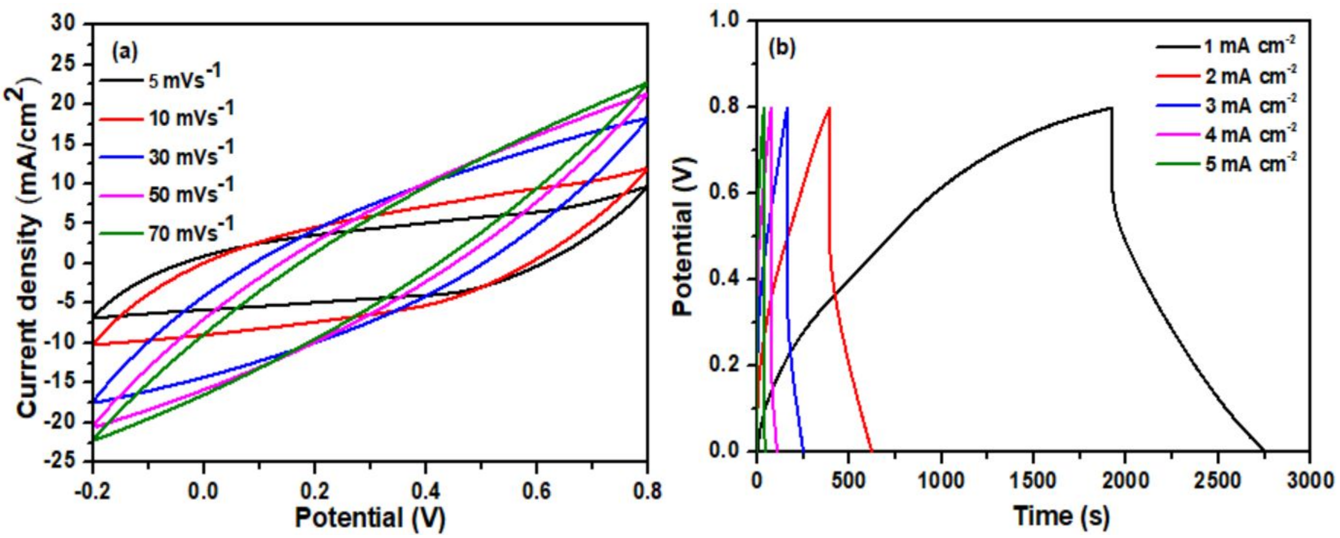

Figure S5 (a) CV plots at different scan rates and (b) GCD plots at different current densities of the VE73/DA/PPy-90 electrode.

Table S1 Capacitive performances of PVA-based hydrogel electrodes reported previously in the literature and in this present study

Electrode materials	Areal capacitance of electrode (C_a)	Specific capacitance of electrode (C_s)	Cycling stability	Reference
PVA/Pectin/PDA/ PPy	1575.7 mF/cm² at a current density of 1 mA cm^{-2}	262.6 F g^{-1} at a current density of 0.17 A g^{-1}	70% after 10,000 GCD cycles	This study
PANI/PVA	602 mF cm^{-2} at 1 mA cm^{-2}	351.0 F g^{-1} at a current density of 0.1A g^{-1}	85.7% after 1000 GCD cycles	(1)
PANI@$Ti_3C_2T_x$/ PVA	103.8 mF cm^{-2} at 2 mA cm^{-2}	-----	90% after 10,000 GCD cycles	(18)
PPy/PVA	----	216.4 F g^{-1} at a current density of 1A g^{-1}	94% after 10,000 GCD cycles	(32)
MnO_2/PANI/PVA	------	293.0 F g^{-1} at 10 mV s^{-1}	81.0% after 1000 GCD cycles	(33)
GO/PEDOT/PVA	------	281.2 F g^{-1} at a current density of 0.1A g^{-1}	86.4% after 10,000 GCD cycles	(34)
PACP/PVA	1267 mF cm^{-2} at 1 mA cm^{-2}	633.5 F g^{-1} at a current density of 0.5A g^{-1}	86.4% after 10,000 GCD cycles	(35)
PEDOT/PVA	335.4 mF cm^{-2} at 10 mV s^{-1}	-----	------	(36)
PEDOT/PVA	128.9 mF cm^{-2} at 0.5 mA cm^{-2}	----	98.1% after 1,000 GCD cycles	(37)
PPy/PVA	224 mF cm^{-2} at 0.8 mA cm^{-2}	----	----	(38)
